# Expression Pattern of *Axin2* During Chicken Development

**DOI:** 10.1371/journal.pone.0163610

**Published:** 2016-09-28

**Authors:** Gesa Eckei, Marion Böing, Beate Brand-Saberi, Gabriela Morosan-Puopolo

**Affiliations:** Department of Anatomy and Molecular Embryology, Ruhr-University of Bochum, Bochum, Germany; Imperial College London, UNITED KINGDOM

## Abstract

Canonical *Wnt*-signalling is well understood and has been extensively described in many developmental processes. The regulation of this signalling pathway is of outstanding relevance for proper development of the vertebrate and invertebrate embryo. *Axin2* provides a negative-feedback-loop in the canonical *Wnt*-pathway, being a target gene and a negative regulator. Here we provide a detailed analysis of the expression pattern in the development of the chicken embryo. By performing *in-situ* hybridization on chicken embryos from stage HH 04+ to HH 32 we detected a temporally and spatially restricted dynamic expression of *Axin2*. In particular, data about the expression of *Axin2* mRNA in early embryogenesis, somites, neural tube, limbs, kidney and eyes was obtained.

## Introduction

*Axin2* (also called Axil or Conductin) is a homologue of Axin protein. It shares approximately 45% of amino acids with Axin [1, 2]. The Axin protein houses four highly conserved functional domains throughout the species [3–5]. The N-terminal RGS-domain has been found to interact with the tumour suppressor gene adenomatous polyposis coli (APC) [1, 6]. Central in the Axin protein, binding sites for *β*-catenin and for the glycogen-synthase kinase 3 beta (GSK-3*β*) were described [4]. At the C-terminal region, the DIX-domain is located that resembles the sequence of a DIX—domain in dishevelled protein (Dvl) and promotes its interaction with Axin [7]. At its C-terminus, Axin also interacts with the protein phosphatase 2A (PP2A) [3, 8, 9]. Being responsible for the degradation of the downstream canonical *Wnt*-signalling pathway molecule *β*-catenin, Axin and *Axin2* function as negative regulators of the canonical *Wnt*-signalling pathway [5, 10–12]. The *Wnt*-signalling pathway is one of the best elucidated signalling pathways. First, the canonical *Wnt*-pathway was described, followed by at least two non-canonical pathways. The pathway of planar cell polarity (PCP) and the *Wnt*/*Ca*^2+^-pathway are referred to as non-canonical pathways [13–15]. These are described to establish orientation in epithelia (PCP) and to play a role in early embryonic ventral patterning (*Wnt*/Ca^2+^-pathway) ([16] for review). Canonical and non-canonical *Wnt*-signalling are known to be enmeshed with each other, as their members partially contribute to more than one pathway [17, 18] and several *Wnt* ligands were described to activate both canonical and non-canonical pathways [19–22]. However, in this study, only the canonical pathway is of relevance, constituting a venue for the Axin family [10]. Central to canonical *Wnt*-signalling stands the transcriptional activator *β*-catenin. When entering the nucleus, *β*-catenin can displace transcriptional repressors such as Groucho [23] from the TCF/LEF transcription factor, which now activates the transcription of target genes ([24] for review). In the unstimulated cell, a multi-protein complex mediates the degradation of *β*-catenin via the ubiquitin proteasome pathway [25, 26]. For this purpose, *β*-catenin is phosphorylated by one of the two kinases of the complex, GSK-3*β* or the casein kinase 1 (CK1).Axin acts as a central scaffold protein in the degradation complex by binding and thus bringing together all important components [3]. For this purpose, Axin and *Axin2* contain highly conserved regions. GSK-3*β* phosphorylates *β*-catenin, which subsequently is ubiquitinated by the E3 ubiquitinase *β*TrCP and degraded by a proteasome [4, 27]. GSK-3*β* further phosphorylates Axin itself, leading to stabilization of its interaction with *β*-catenin [4]. APC and one of the relevant receptors in *Wnt* pathway, the low density lipoprotein related receptor LRP 5/6 are also known to be substrates of GSK-3*β* [28, 29]. Although GSK-3*β* is capable of phosphorylating *β*-catenin alone, Axin unites both proteins, facilitating and significantly accelerating this process [30]. Finally, this process represses *β*-catenin in the cytoplasm to a level that prohibits its access to the nucleus. In the presence of APC, the turnover of *β*-catenin increases [6, 31]. Previous studies suggest a role for APC in the recruitment of several *β*-catenin molecules to the environment of the destruction complex [5, 32, 33]. Upon arrival of *Wnt*-ligands, they bind to the seven-pass transmembrane receptor Frizzled and to its co-receptor, the low density lipoprotein related protein receptor (LRP) 5 or 6 [34–36]. This heterodimeric complex binds Dvl at the cytoplasmatic tail of Fz [37] and initiates the recruitment of Axin and kinases (Gsk3b or Ck1) to the membrane, mediating the dissociation of the *β*-catenin-destruction complex [29, 38, 39]. Several studies have been performed in order to elucidate the exact molecular scenario after *Wnt*-binding. For instance, *Wnt* was described to induce Dvl, that is thought to recruit Axin-bound GSK-3*β* to the membrane, where the latter phosphorylates LRP 5/6 and as a consequence dissociates from Axin [29, 40]. The phosphorylation of LRP 5/6 can equally be achieved by CK1 [39, 41]. Axin priorly was dephosphorylated by another member of the *β*-catenin degradation complex, the protein phosphatase 1 (PP1) [42, 43]. Unphosphorylated Axin releases *β*-catenin [30, 40] and easily binds to LRP 5/6. Binding of Axin to Dvl that is connected to the cytoplasmatic tail of Frizzled, is proposed to facilitate this initial recruitment [27]. Previous studies have further proposed a model for the formation of so called signalosomes, built from multiple associated LRP/Axin complexes [44]. As a result of the dissociation from the *β*-catenin-destruction complex, *β*-catenin is not degraded any more, cytoplasmatic levels rapidly rise and it enters the nucleus [37]. A special function for *Axin2* was found, when discovering its transcriptional dependence on TCF/LEF motive [45]. *Axin2* expression therefore is initiated by canonical *Wnt*-signalling and provides a negative-feedback loop [27, 37, 45]. As this study aims to emphasize the relevance of *Axin2* in regulating the *Wnt*-signalling pathway, it is important to mention the state of the art regarding the role of canonical *Wnt*-pathway in development and in disease. During development, canonical *Wnt*-signalling is described to be required for proper posterior axis formation and for the formation of the head [46–48]. Moreover, *Wnt*-signalling is known to be indispensable in the developing central and peripheral nervous system [49]. It is described to be involved to the segmentation clock during somitogenesis [50] and in the development of several other structures and organs, such as the limbs, the kidney, the gastrointestinal system, the sensory organs and the lungs ([37, 49] for review). In the adult, deregulation of the *Wnt* pathway cause several cancers and *Wnt*-signalling is required for stem cell self renewal [51, 52]. The regulation of *Wnt*-signalling via Axin and *Axin2* impacts embryonic development and health in the adult, as described by many studies. Axin mutant mice failed to survive [11, 53] and display severe developmental defects. Mice with homozygous mutations in *Axin2* developed a secondary caudal body axis [11] and exhibited malformations of the skull due to premature fusion of cranial structures [54]. This malformation is an equivalent to the human disease craniosynostosis, that is described to develop on the basis of *Axin2* mutations [55]. Another developmental defect associated with *Axin2* abnormalities in mice and human is familial tooth agenesis and oligodontia [56, 56, 57]. Further, Axin is related to hepatocellular cancer [58, 59], ovarian cancers [60] and to medulloblastomas [61]. *Axin2* mutations play a secondary role in familal adenomatous poliposis coli (FAP), when the causal mutation is not situated in APC and because proper function of APC requires Axin [62, 63]. Predisposition to colorectal cancer, when carrying mutations in *Axin2* is described [58, 63]. Shedding similar functions than Axin, *Axin2* was previously tested on its functional redundancy [54]. *Axin2* was shown to be able to at least partially compensate for mutated Axin when expressed in the respective cells. Axin however, is expressed in small amounts in all embryonic tissues, while *Axin2* expression was described to be restricted and dynamic during mouse development [11, 45]. Interestingly, Axin was described to be the limiting factor in *Wnt* regulation, referring to its low cytoplasmatic levels [64]. *Axin2* on the other hand, is highly expressed, suggesting an extensive role for *Axin2* regulation in certain tissues. This observation, together with the fact that *Axin2* is a target of *Wnt*-signalling, indicates the importance of *Axin2* mediated negative regulation in certain tissues. In this study, we demonstrate the dynamic expression pattern of *Axin2* in the development of the chick.

## Materials and Methods

### Embryos

Fertilized eggs of Gallus gallus domesticus were incubated at 37^°^*C* and 80% relative humidity. Eggs were provided by a local breeder (Sörries-Trockels Vermehrungszucht). Staging was performed according to Hamburger and Hamilton [65].

The obtained chicken embryos were isolated, fixed in 4% PFA for at least 24*h*. For description and analysis of the expression pattern of *Axin2* during chicken development, chicken embryos in developmental stages HH 04 to HH 32 were proceeded in *in-situ* hybridization.

### Whole mount *in-situ* hybridization

Whole mount *in-situ* hybridization was performed as previously described [66], using c*Axin2* riboprobe for detection of *Axin2* transcripts in all embryonic tissue.

#### Generation of a riboprobe for *in-situ* hybridization

The probe for c*Axin2* *in-situ* hybridization was generated from a pCMS-EGFP plasmid containing a full length *Axin2* coding sequence. It was restricted using EcoRV and SmaI to obtain a 835bp fragment binding from bp926 to bp1788 on *Axin2* mRNA (NCBI Reference Sequence: NC_006105.4). The purified fragment was blunted and cloned to pJET1.2/blunt Cloning Vector. From here, the fragment was excised using XbaI and XhoI and ligated to pBluescript II KS+ Vector. The obtained plasmid was suitable for generating a riboprobe in *in vitro* transcription.

### Sectioning

#### *Vibratome*-sections

The embryos were embedded in 2, 5 − 4% agarose gel and sectioned with *Vibratome* (Leica VT 1000 S) to 50–80*μm*. Sections were collected and covered with cover slips and Aquatex (Merck).

#### *Cryo*-sections

*In-situ* hybridized chicken embryos were embedded in Leica tissue freezing medium^®^and frozen with liquid nitrogen. Obtained blocks were sectioned with Leica CM3050 S *cryo-stat*. Sections were collected on slides, dried and covered using Aquatex (Merck).

#### Ethic statement

According to German legislation, the use of embryonic vertebrates in an animal experiment needs approval only if the animal is in the last third of its embryonic development. In the case of chicken, this means that experiments done on animals before embryonic day 14 (E14) are not regarded as an animal experiment by the Tierschutzgesetz, and therefore, do not need approval or governmental permission.

The chicken embryos sacrificed for this work were between developmental stages HH+04 (E1) and HH32 (E7.5). All embryos were sacrificed at the end of the study by opening the shell and tearing the allantois and amnion with forceps. Thereafter, the embryos were immersed in 4% PFA/PBS solution for fixation. No permits were required for the described study, which complied with all relevant regulations.

## Results and discussion

### 0.1 Expression pattern of c*Axin2* during early chicken embryogenesis

**Fig 1:**
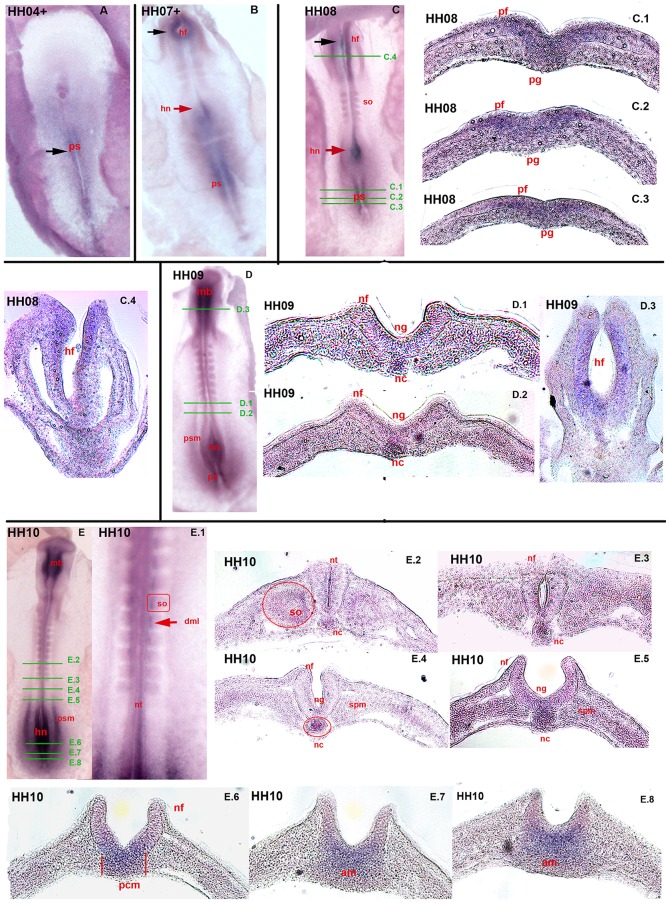
Expression of c*Axin2* in stages HH 04+ to HH 10. Overviews and transversal sections of chicken embryos. Green bars in overviews (C, D, E) indicate sectioning level. (A) HH 04+: *Axin2* transcripts in *ps* (black arrow). (B) HH 07+: expression intensified in *ps*, *hn*(red arrow) and *hf* (black arrow). (C) HH 08: expression in *hf* (black arrow), *hn* (red arrow) and *ps*. (C.1) *Axin2* expression in *pf* and *pg*. (C.2) intense staining in the *pf*. (C.3) expression thickened ectoderm as a first step of neurulation. (C.4) transcripts in the most medial inner epithelium of the *hf*. (D) HH 09: expression in *hn*, faintly in the *psm* and in the *mb*. (D.1, D.2) expression in the *ng* and in the *nc*. (D.3) strong expression in the medial layer of the *hf*. (E) HH 10: strong *Axin2* expression in *hn* and *psm*, as in the *mb*. (E.1) higher magnification of the *so* and *nt* shows expression in the medial *so*, the *dml*. (E.2) faint staining in medio-dorsal epithelium of the early *so* and in the developing *nt*. (E.3) transcripts rarely detectable in the *nf* prior to closing. (E.4) expression of *Axin2* in the *nc* and in the annealing *nf*. (E.5) expression in the centre of the *nf* and in the *nc*. (E.6, E.7, E.8) upheaval of the *nf* in the distal-most *hn*. (E.6) expression in the centre of the future *nt* and in the *pcm*. (E.7, E.8) transcripts in the folding neuroectoderm expanding towards the *am*. ps-primitive streak, hn-Hensen′s node, hf-head fold, pf-primitive folds, pg-primitive groove, psm-presomitic mesoderm, mb-mid-brain, ng-neural groove, nc-notochord, so-somites, nt-neural tube, nf-neural fold, spm-segmental plate mesoderm, pcm-prechordal mesoderm, am-axial mesoderm.

After whole mount *in-situ* hybridization, a dynamic expression pattern of *Axin2* was found from stages HH 04 to 32. In early embryogenesis, *Axin2* expression was observed in the primitive streak (ps)(Fig 1, A black arrow, B, C, D) and in the Hensen’s node (hn)(Fig 1, B red arrow, C red arrow, D, E). Additionally, the head fold (hf) heavily expresses *Axin2* from stage HH 07+ onwards (Fig 1, B, C black arrows). During further development, in stage HH 10, *Axin2* transcripts were detectable in the Hensen′s node (hn), posterior presomitic mesoderm (psm) (Fig 1, E) and medially in the freshly segmented paraxial mesoderm (dml-dorso-medial lip) (Fig 1, E.1 red arrow). Transversal sections were performed to analyse the expression of *Axin2* during early embryogenesis in detail. They present gastrulation and neurulation processes, where the maturation can be observed in a cranial to caudal axis. The green bars in the whole mount specimens indicate the sectioning level. Sections of HH stage 08 (Fig 1, C.1, C.2, C.3) show the caudally regressing primitive streak (ps) with the primitive groove (pg). The primitive folds (pf) of the ectoderm and the developing mesoderm underlying the primitive groove (pg) express *Axin2* (Fig 1, C.1, C.2, C.3). Further, the transversal section of the head fold (hf) in HH stage 08 (C.4) shows intense expression of *Axin2* in medial parts, facing towards the lumen of the anterior neuropore. In HH 09, during the primary neurulation process, cranially to Hensen’s node (hn) (Fig 1, D, D.1, D.2), only little *Axin2* is expressed in the neural groove (ng) (Fig 1, D.1, D.2) and in the elongating notochord (nc) (Fig 1, D.1 and D.2). At this stage the head folds (hf) at mid-brain (mb) level have converged (Fig 1, D.3) and *Axin2* expression is increased in the medial neural folds. In Fig 1, E.2, E.3, E.4 and E.5 (HH 10), the segmental plate mesoderm (spm) is formed, as the neural folds (nf) extend distally to form the neural tube (nt). *Axin2* is expressed in the neural groove (ng) and in the notochord (nc)(Fig 1, E.4, E.5). At HH stage 10 more cranially, first somites (so) are shaped in the segmental plate mesoderm (Fig 1, E.2, E.3), as the neural folds (nf) fuse to form the neural tube (nt). In sections E.6 to E.8 (Fig 1), the development of the caudally shifted Hensen’s node (hn) is depicted. *Axin2* expression is restricted to the central Hensen’s node (Fig 1, E.6, E.7, E.8) expanding towards the ventral axial mesoderm (am). In picture E.6 (Fig 1), the prechordal mesoderm (pcm) is heavily stained for *Axin2*.

**Fig 2:**
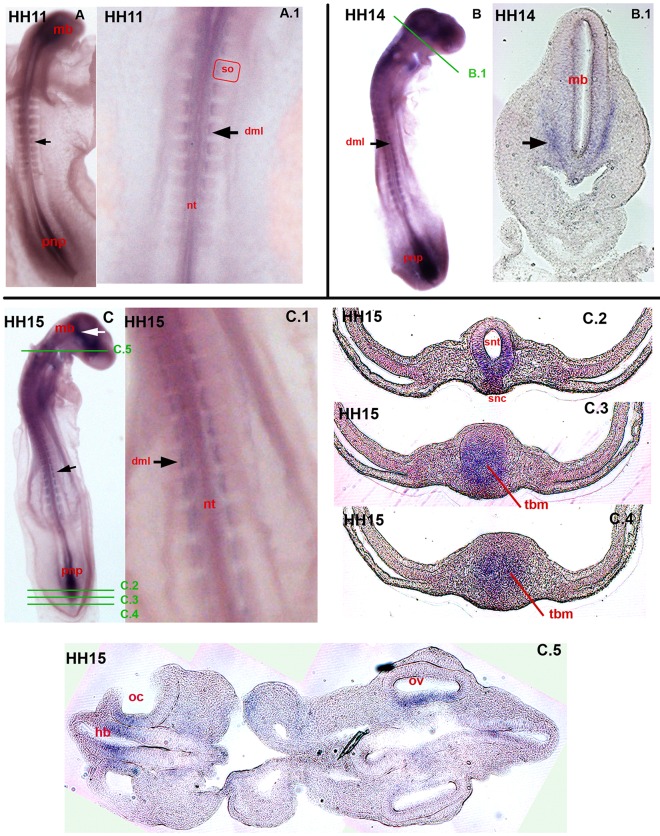
Expression of c*Axin2* in stages HH 11 to HH 15. Overviews (A, B, C) and transversal sections of chicken embryos. Green bars in overviews indicate sectioning level (C). (A) HH 11: *Axin2* expression in the head, *pnp*, *nt* and (A, A.1) *dml* of the *so* (black arrow). (B) HH 14: strong expression in the *dml* (black arrow). (B.1) transversal section through the head at mid-brain level with transcripts in the medial head fold in in the adjacent neighbouring mesenchyme (black arrow). (C) HH 15: *Axin2* expression in the brain (white arrow), the *dml* (C.1 black arrow) and in the *pnp*. (C.2) expression in the *snc* and in the ventral *snt*. (C.3, C.4) *Axin2* expression in the *tbm*. (C.5) section through head and neck with expression of *Axin2* in the *oc*, the neighbouring *hb* and the *ov*. pnp-posterior neuropore, nt-neural tube, dml-dorso-medial lip, so-somites, snc-secondary notochord, snt-secondary neural tube, tbm-tail bud mesoderm, oc-otic cup, hb-hind-brain, ov-optic vesicle.

By stage HH 11, the expression of *Axin2* in the dorso-medial lip (dml) appears (Fig 2, A, A.1 black arrows). This expression intensifies as the somites mature (Fig 2, HH 14: B, black arrow and HH 15: C, C.1 black arrows). Additionally, the posterior neuropore (pnp) is intensively stained for *Axin2* (Fig 2, A, B, C). Regarding the head of the depicted embryos in Fig 2, *Axin2* expression is visible predominantly in the mid-brain (mb)(Fig 2, A, B.1, C white arrow). During secondary neurulation, which describes the elongation of the neural tube (nt) into the tail bud, *Axin2* is expressed centrally in the tail bud mesoderm (tbm)(Fig 2, C.2, C.3, C.4) in HH 15 and ventrally in the recently formed secondary neural tube (snt) and secondary notochord (snc)(Fig 2, C.2).

In stage HH 14 at mid-brain level (Fig 2, B.1), the anterior neuropore has closed and *Axin2* expression has shifted to a patch in the ventral mesoderm, flanking the mid-brain (mb)(Fig 2, B.1 black arrow). In HH stage 15, *Axin2* expression is detectable in the developing sensory organs, eye and ear, for the first time (Fig 2, C.5). *Axin2* mRNA was detected in the otic cup (oc)(Fig 2, C.5) and adjacent hind-brain (hb), as well as in the out-pocketing optic vesicle (ov)(Fig 2, C.5). The optic vesicle (ov) forms laterally from the prosencephalon, where *Axin2* is transcribed in the medial wall.

Previous studies have investigated the role of *Wnt*-signalling during gastrulation, neurulation, axis- and head formation. In the early patterning events of the vertebrate body, canonical *Wnt*-signalling is believed to first act as dorsalizing and later as posteriorizing signal [67, 68]. In concordance to that, several *Wnt*-mutant mice exhibit truncated posterior axis, lost tail formation and disturbed somitogenesis [69, 70]. Experiments in chicken and Xenopus resulted in axis duplication and disturbed head formation after *Wnt* overexpression [71]. Proper formation of the head requires *Wnt* inhibition in the anterior embryonic tissue [72–74]. Ectopic expression of *Wnt* inhibitors was found to induce notochord formation [75]. The examination of Axin knockouts revealed its function in ventralizing the respective tissue and in inhibiting posterior axis formation [11]. Furthermore, Axin loss of function in Xenopus resulted in disturbed closure of neural folds, head folds and the duplication of the allantois [76]. These findings together with the our new observed expression of *Axin2* during chicken embryogenesis support the idea that appropriate regulation *Wnt*-signalling via *Axin2* influences body patterning, axis elongation and head formation. The expression of several *Wnts* in the chicken primitive streak and Hensen’s node reinforce this hypothesis [77].

### 0.2 Expression pattern of c*Axin2* in stages HH 17 to 32

**Fig 3:**
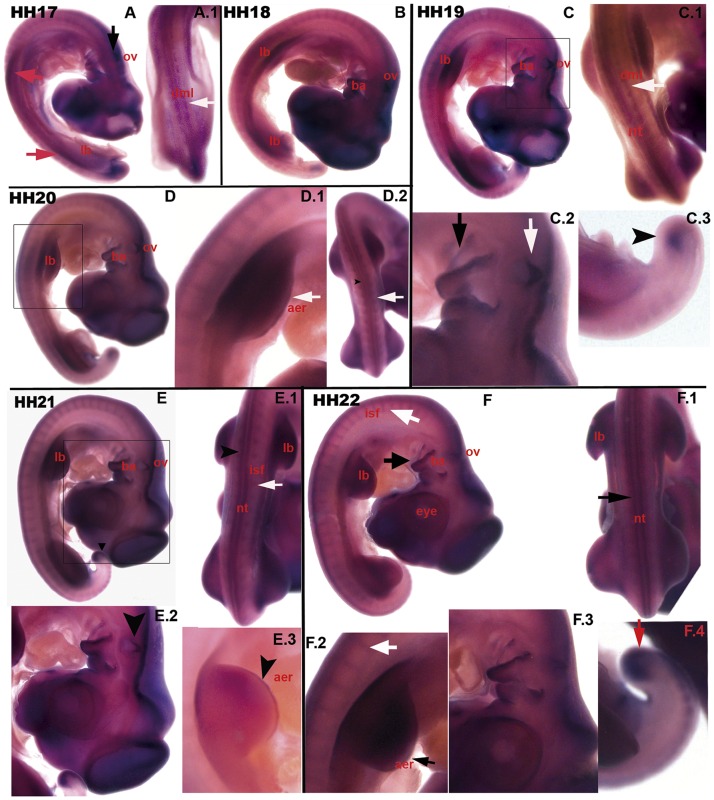
Expression pattern of c*Axin2* from HH stage 17 to 22. (A) HH 17 embryo with *Axin2* expression in the brain, *lb* (red arrows), tail and *ov* (black arrow). (A.1) dorsal view: expression in the *dml* (white arrow). (B) HH 18: more prominent staining in the *lb* and in the *ba*. (C, C.1, C.2 & C.3) HH 19: *Axin2* transcripts in the *nt*(C.1) and *dml* (C.1, white arrow), in *lb* (C, C.1), in *ov* (C.2, white arrow), *ba* (C.2, black arrow) and in the tip of the tail (C.3, black arrow). (D, D.1, D.2) HH 20: similar expression of c*Axin2*. (D.1) the wing bud expresses *Axin2*, white arrow: *aer*. (D.2) dorsal view: prominent expression in *nt* (black arrowhead) and *isf*(white arrow). (E, E.1, E.2, E.3) HH 21: Transcripts in the tip of the tail (E, black arrowhead), in *nt* and *so*(E.1), in the otic anlage (E.2, black arrowhead) and in the developing *lb* (E.3). (F, F.1, F.2, F.3 and F.4) HH 22: consecutive expression of *Axin2* mRNA in *ba* (F, black arrow; F.3), *nt* (F.1, black arrow), *isf* (F, white arrow, F.2, white arrow) and tail (F.4, red arrow). lb-limb buds, ov-otic vesicle, dml-dorso medial lip, ba-branchial arches, nt-neural tube, aer-apical ectodermal ridge, isf-intersomitic furrow, so-somites.

At HH stage 17, the chicken limb buds (lb) are distinguishable, expressing *Axin2* mRNA from their onset (Fig 3, A red arrows). During the rapid outgrowth of the limb buds (lb) the *Axin2* expression increases (Fig 3, HH 19: C, HH 20: D.1, HH 21: E.3, HH 22: F.2). The apical ectodermal ridge (aer) is notably stained (Fig 3, D.1 white arrow, E.3 black arrowhead, F.2 black arrow). In somites (so), *Axin2* expression shifts from the medial somite to the intersomitic furrow (isf)(Fig 3, HH 21: E.1 white arrow; HH 22: F, F.2 white arrows). After whole mount *in-situ* hybridization, the neural tube (nt) is stained for *Axin2* in two longitudinal stripes, at first weakly (Fig 3, HH 19: C.1, HH 20: D.2 black arrowhead), then stronger (Fig 3, HH 21: E.1 black arrowhead, HH 22: F.1 black arrowhead). Moreover, the mesenchyme of the sprouting tail bud (tb) expresses *Axin2* (Fig 3, HH 17: A, HH 19: C.3 black arrowhead, HH 22: F.4 red arrow). As well as at other expression sites, *Axin2* transcription relatively increases during maturation of the respective tissue or organ. At the head region, *Axin2* is expressed in the otic vesicle (ov)(Fig 3, HH 17: A black arrow; HH 19: C.2 white arrow; HH 21: E.2 black arrowhead). Furthermore, the branchial arches (ba) are specifically stained after *Axin2* *in-situ* hybridization (Fig 3, HH 19: C.2 black arrow, HH 21: E.2, HH 22: F.3). Moreover, the brain vesicles express *Axin2*.

**Fig 4:**
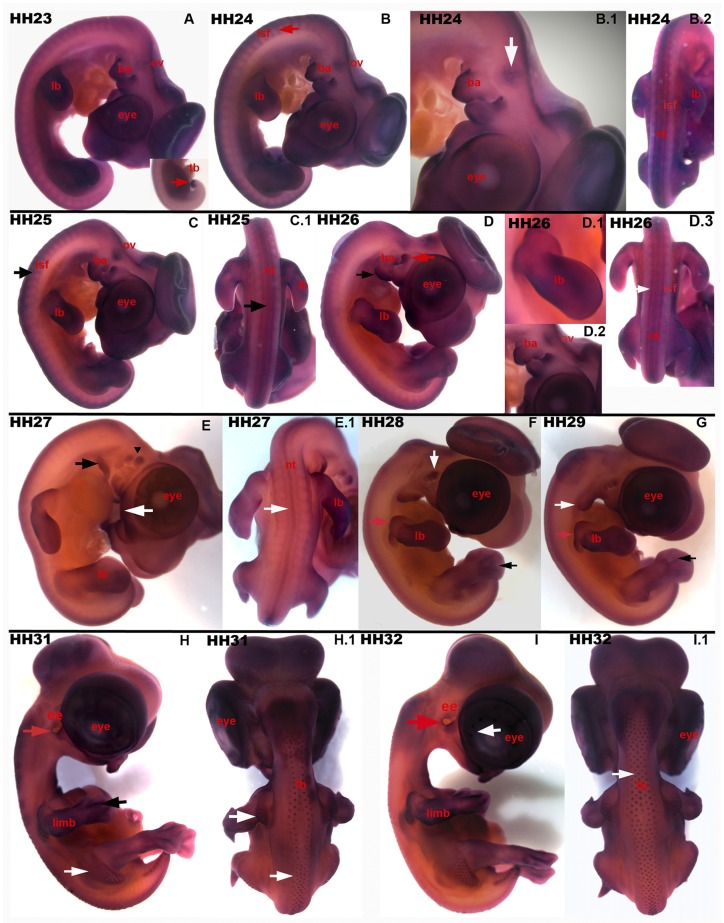
*Axin2* transcripts in chicken embryos from stage HH 23 to HH 32. (A) HH 23: expression pattern resembles what is described in Fig 2. Limbs, inner ear, brain, eye, and *tb* express *Axin2* (A). (B, B.1 & B.2) HH 24: *Axin2* expression in the *ov* (B.1, white arrow) and *ba* (B.1), as well as in *nt* and *isf*(B.2, dorsal view). (C, C.1) HH 25: *Axin2* is expressed at similar embryonic structures with little change. (D, D.1, D.2, D.3) HH 26: *Axin2* expression in *ba* (D, black and red arrow; D.2), *ov*(D.2), *lb*(D.1), brain (D), eye (D), *isf* (D.3, white arrow) and *nt* (D.3). (E) HH27: *Axin2* transcripts in the facial whilst (E, white arrow), in the *ba* (black arrows), as well as in *lb*. The dorsal view (E.1) expression in the *nt* (white arrow). *Ba* display specific staining for c*Axin2* (HH 28: F & HH 29: G, white arrows). HH 28 and 29: intense expression in the embryonic shoulder (F & G, red arrows). Expression of *Axin2* in the forming interdigital spaces (F & G, black arrows). (H, H.1) HH 31: expression of *Axin2* mRNA in *lb* and apoptotic interdigital zones (H, black arrow), at the *ee*(H, red arrow) and in the *fb*(H & H.1, white arrows). (I, I.1) HH 32: *Axin2* transcripts in the eye (I, white arrow), *ee* (I, red arrow) and in the *fb* (I.1, white arrow). lb-limb buds, ov-otic vesicle, ba-branchial arches, tb-tail bud, nt-neural tube, isf-intersomitic furrow, fb-feather buds, ee-external ear.

HH 23 to 29 embryos (Fig 4) express *Axin2* in similar regions, compared to the earlier developmental stages, with little changes. *Axin2* is expressed in brain and otic vesicle (ov) throughout these stages (Fig 4, HH 24: B.1 white arrow). In addition, the branchial arches (ba) show intense staining (Fig 4, HH 24: B.1), which becomes restricted during development and predominantly was observed on the protuberances of the mandibular (Fig 4, HH 27: E black arrowhead, HH 28: F white arrow) and maxillary arch, respectively (Fig 4, HH 29: G white arrow). The expression pattern in the neural tube (nt) changes from two longitudinal lines (as described above) to one central line (Fig 4, HH 26: D.3; HH 27: E.1 white arrows). Another expression site of *Axin2* is presented in Fig 3, picture E. Here, the white arrow indicates an *Axin2* expression in the facial development. *Axin2* is still expressed in the limbs (lb) by stage HH 26 (Fig 4, D.1). Here, it is notable that in further developed stages the future shoulder is heavily stained (Fig 4, HH 26: D.1, HH 28: F and HH 29: G red arrows). The interdigital zones, where programmed cell death occurs, express *Axin2* (Fig 4, HH 28: F and HH 29: G black arrows). This observation was continuously found in the development of digits in later stages (Fig 4, HH 31: H black arrow and HH 32: I). Strong *Axin2* expression is also visible in these older stages’ shoulders (Fig 4, HH 31: H and HH 32: I). The chicken external ear (ee) expresses *Axin2* as well (Fig 4, HH 31: H and HH 32: I red arrows). Finally, *Axin2* is expressed in the first rows of feather buds (fb) on the back of the farthest developed stages (Fig 4, HH 31: H, H.1 and HH 32: I.1 white arrows), as at the shoulders and hips. On the chicken eye, the developing scleral ossicles express *Axin2* (Fig 4, HH 32: I white arrow).

### 0.3 *Axin2* expression during somitogenesis

**Fig 5:**
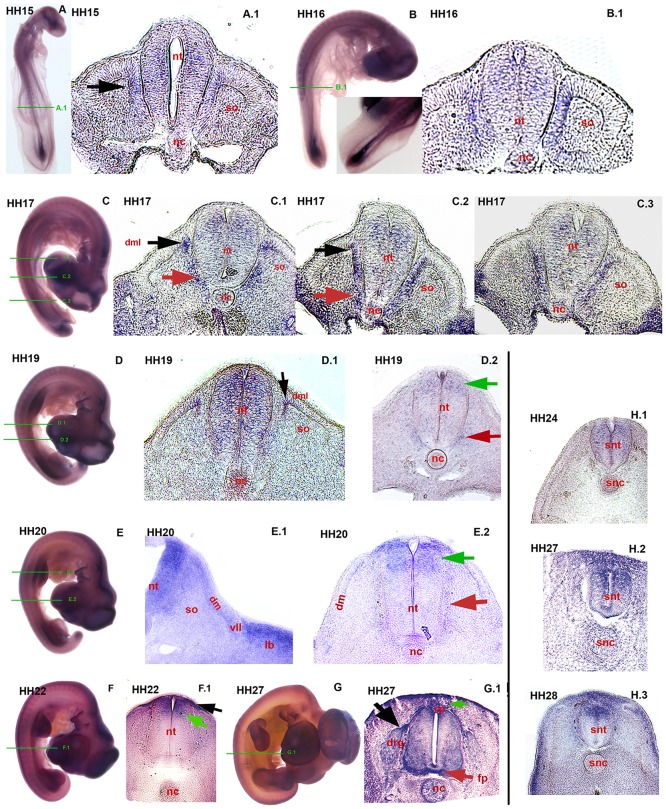
*Axin2* expression during somitogenesis and in the developing neural tube. Transversal sections: green bars in overviews indicate sectioning level. (A) HH 15, overview; (A.1) expression in medial epithelial *so*(black arrow) and faintly all over the *nt*. (B) HH 16, overview; (B.1) increased expression in the medial somitic epithelium. (C) HH 17, overview; (C.1) *Axin2* in *dml* (black arrow), in the mesenchyme ventrally flanking the *nt* (red arrow) and predominantly in the dorsal *nt*. (C.2) Expression in dorso-medial somitic epithelium (black arrow), ventrally in the mesenchyme (red arrow) and all over the *nt*. (C.3) Expression in the medial epithelial *so*. (D) HH 19, overview; (D.1) expression in *dml* and *nt*; (D.2) expression restricted to dorsal *nt* (green arrow) and ventrally in the neighbouring tissue (red arrow). (E) HH 20, overview; (E.1) expression throughout the *dm*; (E.2) *Axin2* transcripts in the dorsal *nt* (green arrow), in the ventro-medially adjacent mesenchyme (red arrow) and weakly in the *dml*. (F) HH 22, overview; (F.1) *Axin2* in the dorsal-most *nt* (green arrow) and in the overlying ectoderm (black arrow). (G) HH27, overview; (G.1) *Axin2* expression in *rp*, *fp* (red arrow), *drg* (black arrow) and subectodermal space (green arrow). (H.1, H.2, H.3) *snt* of the tail. (H.1) HH 24, faint expression in all parts of the *snt*; (H.2) HH 27, restricted and intensified expression in the dorsal *snt* and in overlying subectodermal space; (H.3) HH 28, *Axin2* in dorsal most *snt* and subectodermal space. so-somite, nt-neural tube, dml-dorso medial lip, dm-dermomyotome, rp-roof plate, fp-floor plate, drg-dorsal root ganglion, snt-secondary neural tube.

In transversal sections of *in-situ* hybridized chicken embryos, *Axin2* expression was found during somitic differentiation (Fig 5). Green bars in the whole mount specimens (A, B, C, D, E, F, G) indicate the levels, where the sections have been performed. In the segmented paraxial mesoderm, *Axin2* is expressed in the epithelial somites and in the differentiating dermomyotome. At HH stage 15 transcripts are mainly detectable in the medial and medio-dorsal wall of the epithelial somites (Fig 5, A.1 black arrow). This expression gains intensity in stage HH 16 and 17 as the somite (so) maturates (Fig 5, B.1, C.3). More cranially in HH 17, where somites have maturated even further, deepithelialization of the somite (so) has begun (Fig 5, C.2). *Axin2* expression is relatively strong in the remaining medio-dorsal epithelium (Fig 5, C.2 black arrow) and in the mesenchyme ventrally flanking the neural tube (nt)(Fig 5, C.2 red arrow). Further cranially, where the dermomyotome is almost completely formed (Fig 5, C.1), *Axin2* expression was found in the most ventral parts of the forming *dml* of the dermomyotome (Fig 5, C.1 black arrow) and in a patch adjacent to the ventral neural tube (nt)(Fig 5, C.1 red arrow). In stage HH 19, when the dermomyotome is fully established, transcripts are visible in the ventrally facing margin of the *dml*, neighbouring the sclerotome (Fig 5, HH 19: D.1 black arrow). In HH stage 20, at limb level *Axin2* expression is detectable also in the ventro-lateral lip (vll) (Fig 5, E.1 wing level). In further development, this expression gets restricted to the dorsal half of the dermomyotome (dm), the epaxial myotome and appears more faintly (Fig 5, E.2 interlimb level).

In mice *Axin2* expression was found to oscillate in the segmental plate mesoderm and to occupy a central role for the segmentation of the presomitic mesoderm [50, 78]. We were able to detect *Axin2* expression in the posterior *psm* in chicken from stage HH 09 to HH 16 (Figs 1 and 3). In mice, the expression of *Wnt*-genes alternates with the expression of FGFs in the PSM [78], indicating a similar mechanism in chicken. Interestingly *Axin2* mutant mice still undergo segmentation with slight to average deviation [55, 78]. Additionally, *Axin2* transcripts were found during the maturation of the somites. In this process, a network of many different *Wnt*-molecules and other signals is described to play a role. The patterning of the somites is controlled by dorsalizing *Wnt*1 and *Wnt*3a from the dorsal neural tube [79–81], such as *Wnt*6 from the overlying ectoderm [82]. *Wnt*11 was described to maintain the epithelial status of the *dml*, while *Wnt*6 from the ectoderm maintains the epithelial ventro-lateral lip (VLL) [83]. Additionally, it was found that *Wnt*1 and *Wnt*3a are required for the formation of the *dml* [81]. *Axin2* expression in the *dml* and its progenitors (Fig 5) indicate a potential role in the proper development of the *dml* and the deriving dermis. This hypothesis is supported when regarding the expression of *Axin2* in the dermal derived feather buds (Fig 4, H.1, I.1).

### 0.4 Expression pattern in the developing neural tube

Regarding the development of the neural tube, *Axin2* is expressed from neurulation to the differentiated mature neural tube (nt)(Figs 2 and 5). In Fig 5, the maturation of the neural tube (nt) is depicted. First, *Axin2* mRNA was detected in a sprinkled distribution all over the neural tube (nt)(Fig 5, HH 15: A.1, HH 16: B.1, HH 17: C.1 and HH 19: D.1), with an intensified region at the medio-dorsal neuroepithelium (Fig 5, HH 17: C.1, HH 19: D.1). More cranially in HH 19, this expression appears more intense at the dorsal third (Fig 5, D.2 green arrow), while faint sprinkled expression remains in the ventral half of the neural tube (nt)(Fig 5, D.2). By HH stage 20, predominantly the dorsal expression domain increases even more (Fig 5, E.2 green arrow). Further, the faint expression site in the neighbouring tissue at left an right ventral side of the neural tube (nt) expands dorsally (Fig 5, E.2 red arrow). When maturating, the neural tube (nt) expresses *Axin2* strongly in the dorso-medial neuroepithelium (Fig 5, HH 22: F.1 green arrow). Additionally, *Axin2* transcripts are found in the overlying ectoderm and the subectodermal mesenchyme flanking the dorsal neural tube (nt)(Fig 5, HH 22: F.1 black arrow). In HH 27, *Axin2* expression was observed in the dorsal most part of the neural tube (nt) and in the roof- and floor plate (rp)(fp)(Fig 5, G.1). The black arrow in G.1 (Fig 5) reveals to the tip of the dorsal root ganglion (drg) that heavily expresses *Axin2*. Further, the dorsal ectoderm and subectodermal space overlying the neural tube (nt) are intensively stained (Fig 5, G.1 green arrow).

*Axin2* transcripts were also found in secondary neurulation in the tail bud (Fig 2, C.2, C.3, C.4). After secondary neurulation, the differentiating secondary neural tube (snt) heavily expresses *Axin2* (Fig 5, H.1, H.2, H.3). First, this expression is well distributed over the entire neuroepithelium (Fig 5, HH 24: H.1). During maturation, transcripts were observed in HH 27, (Fig 5, H.2) mainly in the dorsal half of the secondary neural tube (snt) as in the overlying subectodermal mesenchyme and ectoderm. By HH 28 the *Axin2* is missing in the ventral two thirds of the secondary neural tube (snt), but is expressed intensively in the dorsal third, such as in the ectoderm and subectodermal mesenchyme (Fig 5, H.3).

During the development and maturation of the neural tube, the establishment of a dorso-ventral axis through ventralizing *Shh* activity versus dorsalizing *Wnt*-signals has been described [84, 85]. The main *Wnt*-genes expressed in the dorsal neural tube and roof plate are *Wnt*1 and *Wnt*3a [84, 86]. These promote neural proliferation [84, 87]. Therefore, after activation of dorsal *Wnt*-signalling in the chick, dorso-ventral patterning of the neural tube was perturbed and mitogenic activity of neural progenitors was increased [88]. *Wnt*1 and *Wnt*3a inhibition in mice, besides incomplete closure of the neural folds, displayed phenotypic alterations throughout the neural tube including partially absent basal-, roof- and floor plates [89]. In addition, *Wnts* have been identified to play a role in ventrally specified neural progenitors [86, 90]. The countless signalling molecules interacting with the *Wnt*-signalling pathway during neural tube maturation imply that *Axin2* expression and its negative-feedback-loop in canonical *Wnt*-signalling impact this neural development and the basic molecular functions will be of special interest in future research.

### 0.5 Expression pattern of c*Axin2* during limb development

**Fig 6:**
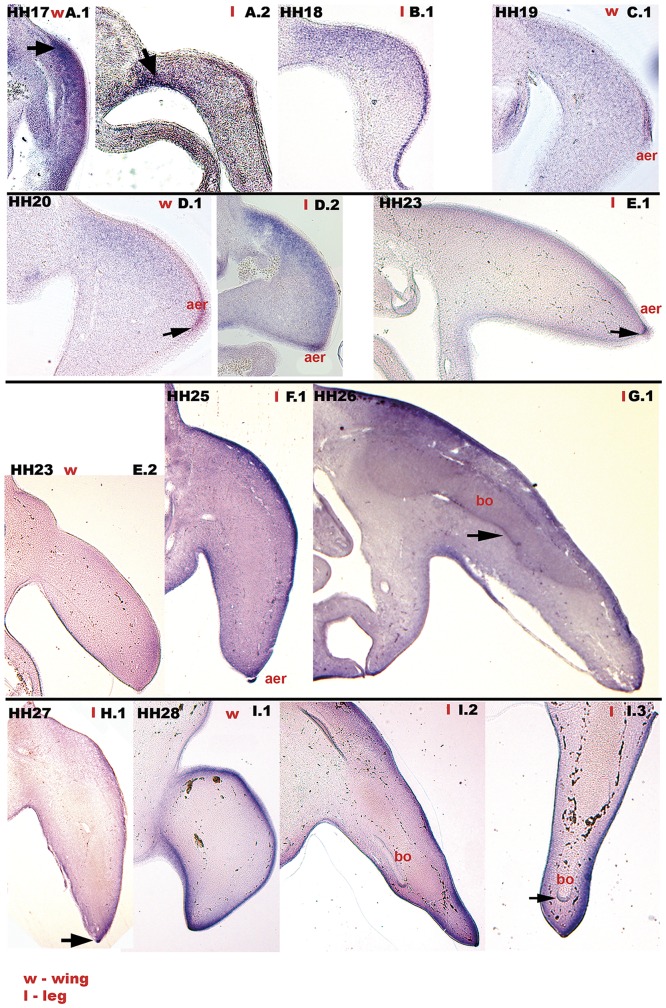
Expression of *Axin2* in chicken limb development. (A.1, wing bud, w) HH 17, strong expression of *Axin2* in the limb, predominantly in the dorsal most part (black arrow). (A.2) The leg bud (l) shows less staining, a patch in the ventro-proximal tissue expresses *Axin2* (black arrow). (B.1, leg bud) HH 18, transcripts in the dorsal mesenchyme and in the distal ectoderm. (C.1) HH 19, expression in the dorsal and most proximal mesenchyme of the wing bud. (HH 19, C.1; HH 20, D.1 black arrow; D.2) *Axin2* expression *aer*. (C.1) HH 19 and (D.1) HH 20, *Axin2* in dorsal subectodermal mesenchyme, in ectoderm and in the *aer*. (E.1, E.2) HH 23; (F.1) HH 25: Transcripts in the ectoderm and *aer* of the limb buds. (E.2–I.3) HH 23 to HH 28: the ectoderm expresses high levels of *Axin2*. (G.1) HH 26, black arrow; (I.3) HH 28, black arrow: the margins of the developing *bo* show transcripts of *Axin2*. aer-apical ectodermal ridge, bo-bones.

Limb development in chicken starts from an out-bulged ridge of the somatic lateral plate mesoderm by stage HH 15. At HH stage 17 the wing bud heavily express *Axin2* predominantly in the dorsal mesenchyme (Fig 6, A.1 black arrow). The hind-limb bud at the same stage is slightly further developed and transcripts of *Axin2* are present in the thickened ectoderm, which gives rise to the apical ectodermal ridge (aer)(Fig 6, A.2), as well as at proximo-ventral margin of the lateral plate mesoderm (Fig 6, A.2 black arrow). In stages HH 18 to HH 20 *Axin2* is expressed in the dorsal mesenchyme of the rapidly outgrowing limb buds (Fig 6, B.1, C.1, D.1, D.2). Moreover, the apical ectodermal ridge (aer) is heavily stained for *Axin2* (Fig 6, B.1, C.1, D.1 black arrow, D.2). By stage HH 23, the transcripts in the dorsal mesenchyme are reduced, though the ectoderm and apical ectodermal ridge (aer) still express *Axin2* (Fig 6, E.1 black arrow). This expression was is consistent in further developed stages (Fig 6, HH 25: F.1; HH 26: G.1; HH 27: H.1 black arrow; HH 28: I.1, I.2 and I.3). Moreover, when regarding the developing bones (bo) in HH stage 26 and 28, we verified *Axin2* mRNA at the marginal perichondrium (Fig 6, G.1 and I.3 black arrows).

Several members of the *Wnt* family are expressed in the developing limb ([37] for review). The outgrowth of the limb bud is mediated by the apical ectodermal ridge (*aer*) [91]. *Wnt* genes are described to initiate the formation of the limb bud (*Wnt*2b) from the lateral plate mesoderm as well as the *aer* (*Wnt*3a) [92]. The *aer* in chicken expresses *Wnt*3a that, by initiating fibroblast growth factor (FGF) expression, mediates the rapid cell proliferation in the mesenchymal progress zone (PZ) underlying the *aer* [93]. Non-canonical *Wnt*7a is expressed in the dorsal ectoderm of the chicken limb, being responsible for dorsalization [94, 95]. Its expression site overlaps an additional expression site for *Wnt*3a in the ectoderm during early limb growth [96]. As *Wnt*7a target genes are expressed in the mesenchyme underlying the dorsal ectoderm, it was suggested that their signalling ranges as far as the target gene expression [97]. We postulate a similar distance of signalling for the canonical *Wnt*3a from early dorsal limb ectoderm as a source for early *Axin2* expression in the dorsal limb mesenchyme. Mutations of *Wnt*3a and *Wnt*7a and FGFs in chicken embryo induced the expression of a gene responsible for a form of polydactyly in human, the Townes-Brock-Syndrome [98]. Later in the limb development, canonical *Wnt*-signalling is described to promote cell proliferation and the differentiation of connective tissue [99]. *Axin2* expression in accordance to our results was reported in the perichondrium of mice [99]. By describing the expression of *Axin2* in the chicken developing limb, we want to reveal its presumable function in regulating *Wnt*-signals that are involved in outgrowth, proliferation and differentiation.

### 0.6 Expression patten of *Axin2* during chicken nephrogenesis

**Fig 7:**
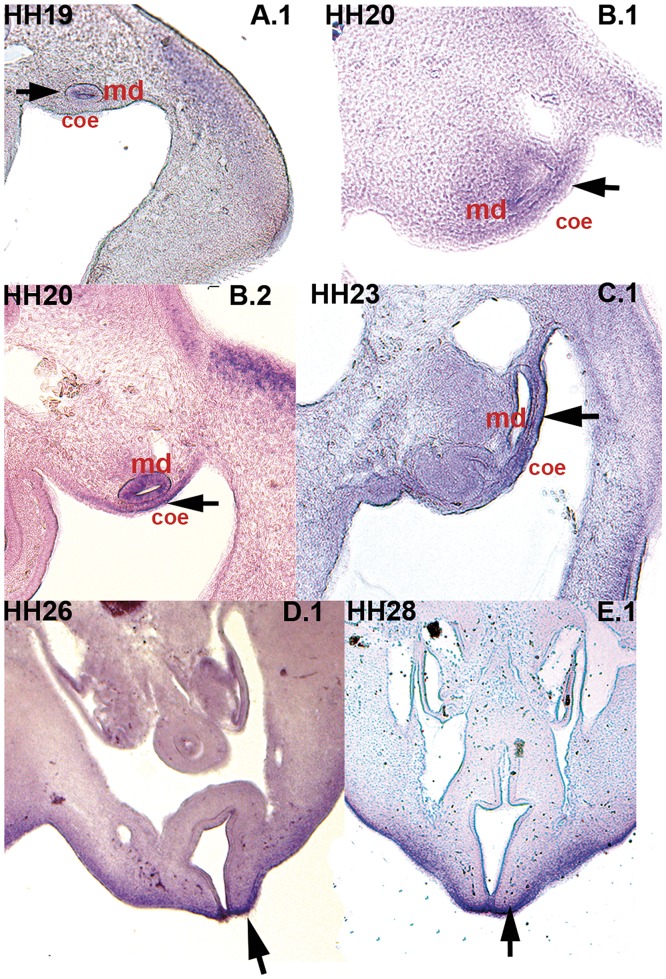
Expression of *Axin2* in nephric duct. *Axin2* mRNA expression in the *md* (HH 19: A:1, leg level, black arrow; HH 20: B.1, interlimb level, black arrow and B.2, leg level; HH23: C.1, caudal interlimb level, black arrow). The overlying thickened ectoderm strongly expresses *Axin2* from HH stage 20 (B.1 & B.2, black arrows). Pictures D.1 and E.1 demonstrate detectable transcripts in the cloacal ectoderm (HH 26: D.1, HH 28: E.1 black arrows). md-mesonephric duct.

The kidney development in birds and mammals takes place in three generations of nephric precursors [100]. In this study, an *Axin2* expression in mesonephric development is described (Fig 7). In stage HH 19 the mesonephric duct (md) at leg level faintly expresses *Axin2* (Fig 7, A.1 black arrow). By HH 20 at interlimb level, the staining expands to the overlying coelomic epithelium (coe)(Fig 7, B.1 black arrow).

At leg level in HH 20, intense *Axin2* expression in the nephric duct (md-mesonephric duct) and coelomic epithelium (coe) is observed (Fig 7, B.2 black arrow). When further differentiating, transcription of *Axin2* decreases, but is still detectable in mesonephric duct (md) and overlying coelomic epithelium (coe)(Fig 7, HH 23: C.1 black arrow). In addition, Fig 7 shows transversal sections of the cloaca, where *Axin2* is expressed predominantly in the coelomic epithelium (Fig 7, D.1, E.1. black arrows).

The role of *Wnt* in the developing kidney has been extensively studied in the past. *Wnt*4 and *Wnt*9b were described to be expressed in the nephric duct and coeloemic epithelium [101–103]. The initiation of tubulogenesis of the developing kidney requires canonical *Wnt*4 and *Wnt*9b signals from the nephric duct [101, 102]. Later in development both *Wnt*-ligands were described to act through the PCP and the Ca2+-dependent pathway as well [104–107]. As the *Wnt*-ligands partially activate different intracellular responses in the course of kidney development, the research faces a challenging aim in understanding this network. In Xenopus a model mediating the switch from canonical to non-canonical *Wnt*-signalling during nephrogenesis was proposed [108, 109]. However, canonical *Wnt*-signalling is known to mediate not only nephron induction, but also its orientation, cell proliferation, specification and differentiation [107, 110–113]. Alterations in canonical and non-canonical *Wnt*-signalling are known to cause polycystic kidney diseases [114, 115]. Taken together, we suggest that *Axin2* might impact kidney development by regulating *Wnt*-signalling as indicated, through its expression in the nephric duct and coelom epithelium. The *Axin2* expression in the coelomic epithelium could possibly hint a role for *Axin2* in the development of the derived Mullerian-duct that develops to form the female genitals. As male gonads develop from the nephric or Wolffian-duct, *Axin2* might be involved in this developmental process as well.

### 0.7 c*Axin2* expression in developing chicken eye

**Fig 8:**
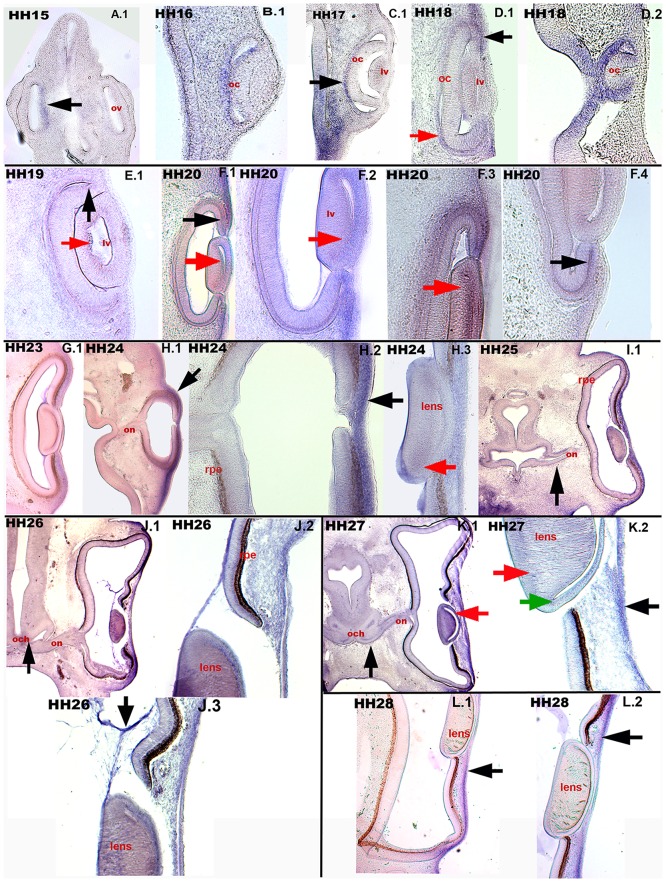
*Axin2* transcripts in optic development. Transversal sections through the developing eye in *in-situ* hybridized embryos. (A.1) HH 15: the primary *ov* expresses *Axin2* in the proximal layer (black arrow). (B.1) HH 16: *Axin2* expression in both layers of the bi-layered secondary *oc*. (C.1) HH 17: *Axin2* expression in the proximal epithelium of the *oc* (black arrow). (D.1) HH 18: *Axin2* expression in the proximal layer of the *oc* (red arrow). The ectoderm surrounding the invaginating, *lv* (black arrow) expresses *Axin2*. (D.2) HH 18: A section through the rostral most part of the *oc*, with highest expression rate in these margins. (E.1) HH 19: the proximal layer of the *lv* expresses *Axin2* (red arrow) as well as the *oc* in both layers adjacent to the vesicular space (black arrow). (F.1, F.2, F.3, F.4) HH 20: expression in the ectoderm surrounding the eye, in the directly underlying mesenchyme, in the epithelium of the *oc* towards the lumen (F.1, black arrow; F.4, black arrow) and in the lens epithelium (F.1, F.2, F.3, red arrows). (HH 23: G.1; HH 24: H.1 and H.2, black arrows; HH 25: I.1; HH 26: J.1, J.2, J.3; HH 27: K.2, black arrow, HH 28: L.1, L.2, black arrows) *Axin2* expression in the ectoderm and subectodermal mesenchyme (future cornea). (HH 25: I.1, black arrow; HH 26: J.1, black arrow, HH 27: K.1, black arrow) c*Axin2* expression in the developing *on* and *och*. (H.3, HH 24; K.2, HH 27) expression of *Axin2* in the developing lens (red and green arrows). ov-optic vesicle, oc-optic cup, lv-lens vesicle, on-optic nerve, och-optic chiasm, rpe-retinal pigmented epithelium.

The chicken eye initially develops, as the prosencephalon out-pockets and the optic vesicle (ov) invaginates to the head mesenchyme. *Axin2* in this process is expressed in the proximal layer of the bi-layered optic vesicle (ov)(Fig 8, HH 15: A.1, black arrow). By stage HH 16 the lens vesicle (lv) has formed from the ectoderm (Fig 8, B.1). *Axin2* transcripts are still detectable mainly in the proximal layer of the optic cup (oc)(Fig 8, B.1, HH 17: C.1 black arrow). An additional expression in the subectodermal mesenchyme overlying the optic cup (oc) and surrounding the lens vesicle (lv) is established at stage HH 18 (Fig 8, D.1 black arrow). While the lens vesicle (lv) expresses little *Axin2* in the inner lens epithelium (Fig 8, HH 19: E.1 and HH 20: F.1, F.2. F.3 red arrows), transcripts in the optic cup (oc) are found in both proximal and distal layer at the epithelial margins facing towards the vesicular space (Fig 8: HH 19: E.1 black arrow, HH 20: F.4 black arrow). In the following observed stages, the proximal layer of the optic cup (oc) has formed the retinal pigmented epithelium (rpe), whereas the distal layer differentiates into the retina [116]. *Axin2* expression was found only in the lens (Fig 8, HH 24: H.3 red arrow), ectoderm and subectodermal mesenchyme covering the eye (Fig 8, HH 24: H.1 and H.2 black arrows). Regarding the formation of the optic nerve (on) and optic chiasm (och), *Axin2* expression is observable in the approaching and fusing neuroepithelial layers (Fig 8, HH 25: I.1, HH 26: J.1, HH 27: K.1 black arrows). Further, *Axin2* is expressed in the future cornea covering the eye (Fig 8, HH 25: I.1, HH 26: J.1, HH 27: K.2 black arrow, HH 28: L.1 and L.2 black arrows) and in the posterior lens epithelium (Fig 8, HH 26: J.2 and J.3; HH 27: K.1 and K.2 red and green arrows).

Anteriorly expressed inhibitors of canonical *Wnt* signals are required for the initiation of the eye as described in zebrafish [117, 118]. Later, *Wnt*2b is expressed in the proliferative lens epithelium [119], retinal pigmented epithelium (rpe) and periphery of the optic cup [120–122]. Further, *Wnt*3 and *Wnt*11 were found to be expressed in the outer layer of the chicken optic cup [122]. *Wnt*2b was described to be responsible for maintaining the proliferative state of neural progenitors in the retina in chick [123]. Previous studies have reported a depigmentation of the retinal pigmented epithelium (rpe) after disruption if *Wnt*2b signalling in the chicken eye [120]. Our observeded *Axin2* expression in the lens overlaps with regions of increased cell proliferation, which express *Wnt*-ligands as well [122, 124, 125]. The chicken developing cornea and corneal stroma cells express *Wnt*3a and *Wnt*9b [126]. Interestingly, a subgroup of the disease familial adenomatous poliposis coli (FAP), which is caused by a truncation in APC or *Axin2*, the Gardner syndrome, includes a congenital hypertrophy of the *rpe* [127]. Additionally, some cases of tetra amelia, which is the result of homozygous *Wnt*3 mutations, exhibit optic malformations [128].

## Conclusion

In the present study, we describe the expression pattern of avian *Axin2* during embryonic development. We found a dynamic, temporally and spatially restricted expression pattern in many developing structures and tissues. In the early development of the chick, *Axin2* was expressed in the primitive streak and underlying mesoderm, in the neural folds and in the head fold. It was additionally expressed during secondary neurulation in the tailbud mesenchyme. Here, the pre-somitic mesoderm as well transcribes *Axin2*. We were able to detect such expression in the posterior *psm* and during the maturation of the somites in its medial epithelium and in the *dml*. By this developmental stage, transcripts were also detectable in the brain and differentiating neural tube. In the developing limb a dynamic expression was found. Furthermore, we detected *Axin2* mRNA in the nephric duct and coelomic epithelium. Regarding the head of the chicken embryo, *Axin2* was expressed in branchial arches and sensory anlagen. Later in development, expression in feather buds, interdigital spaces, external ear and scleral ossicles on the eye was observed.

The expression of *Axin2* in mice was previously found in the primitive streak, head folds, neural tube, branchial arches I and II (maxillary and mandibular arch), *psm* and *dml*, tailbud, limbs, kidney and brain [45, 78].

These findings are mainly consistent to the expression we found in the chick. Additionally, we were able to show *Axin2* expression in the developing eye and in the otic vesicle. With this study we want to point out the often neglected impact of *Axin2* in many *Wnt*-dependant developmental processes. While *Wnt*-ligands are extensively studied, investigating their regulation through *Axin2* in the respective tissues might help understanding the interactions of different signalling factors.
